# Accurate Detection for Zirconium Sheet Surface Scratches Based on Visible Light Images

**DOI:** 10.3390/s23167291

**Published:** 2023-08-21

**Authors:** Bin Xu, Yuanhaoji Sun, Jinhua Li, Zhiyong Deng, Hongyu Li, Bo Zhang, Kai Liu

**Affiliations:** 1 School of Mechanical Engineering, Sichuan University, Chengdu 610065, China; bin_xu@outlook.com (B.X.); sunyhj999@outlook.com (Y.S.); jinhua07@hotmail.com (J.L.); 2 Nuclear Fuel and Material Institute, Nuclear Power Institute of China, Chengdu 610213, China; dengzhy08@163.com (Z.D.); lihongyuzsdyg@163.com (H.L.); 3 College of Electrical Engineering, Sichuan University, Chengdu 610065, China; kailiu@scu.edu.cn

**Keywords:** noise removal, texture suppression, nondestructive testing, scratch detection

## Abstract

Zirconium sheet has been widely used in various fields, e.g., chemistry and aerospace. The surface scratches on the zirconium sheets caused by complex processing environment have a negative impact on the performance, e.g., working life and fatigue fracture resistance. Therefore, it is necessary to detect the defect of zirconium sheets. However, it is difficult to detect such scratch images due to lots of scattered additive noise and complex interlaced structural texture. Hence, we propose a framework for adaptively detecting scratches on the surface images of zirconium sheets, including noise removing and texture suppressing. First, the noise removal algorithm, i.e., an optimized threshold function based on dual-tree complex wavelet transform, uses selected parameters to remove scattered and numerous noise. Second, the texture suppression algorithm, i.e., an optimized relative total variation enhancement model, employs selected parameters to suppress interlaced texture. Finally, by connecting disconnection based on two types of connection algorithms and replacing the Gaussian filter in the standard Canny edge detection algorithm with our proposed framework, we can more robustly detect the scratches. The experimental results show that the proposed framework is of higher accuracy.

## 1. Introduction

Zirconium and its compounds have unique physicochemical properties, e.g., amazing corrosion resistance, extremely high melting point, and ultrahigh hardness and strength, accounting for their applications in engineering and science [1,2]. As a relatively rare metal material, zirconium sheet plays a significant role in machinery manufacturing, aerospace, nuclear reactor, chemical industry, ceramic industry, and other fields [3,4,5,6,7,8]. However, in various production and processing processes, scratches will appear on the surface of zirconium sheets due to various factors. The surface quality of zirconium sheets will directly affect the performance and quality of final products; therefore, it is necessary to detect the location and shape of scratches. At present, the scratches on the surface of zirconium sheets are usually detected manually. However, manual detection has many shortcomings, such as high false detection rate and low efficiency. Therefore, we urgently need a method for scratch detection of zirconium sheets.

At the same time, with the development of modern industry and science and technology, nondestructive testing (NDT) is widely used in various fields, e.g., aerospace, machinery industry, shipbuilding, automobile, etc. [9,10,11,12,13]. NDT technology includes ultrasonic, machine vision, radiographic, eddy current, and other methods. Among them, compared with other methods, machine vision based on image processing technology has great advantages, e.g., automation, high precision, easy operation, etc. Therefore, we choose machine vision to detect scratches on the surface of zirconium sheets [14].

However, due to the interference of the surface features, which are noise and texture of zirconium sheets, it is difficult to use machine vision methods to detect scratches. In other words, noise will mask the details of images, resulting in breakpoints in the extracted scratch contour. In addition, texture will mistakenly become branches of the contour, destroying the size and shape of the contour [15,16,17]. Therefore, removing noise and suppressing texture before scratch detection are key to obtain the region of scratches. Traditional algorithms typically detect targets based on image features, e.g., gradient, color, and texture. Due to that the detection objects are in the complex texture background, the difference between the results obtained from color-based image segmentation and real value is significant [18,19,20]. Researchers from different countries have proposed the following noncolor methods to solve such problems [21,22,23].

Renuka et al. [24] presented an objective calculation method of denoising threshold based on dual-tree complex wavelet transform (DTCWT), which has good edge preservation and denoising ability. Liu et al. [25] presented a nonreference image denoising method based on enhanced DTCWT and bilateral filter. Compared with other algorithms, the results show that the denoising effect of this method is better than other methods. Li et al. [26] proposed a you only look once (YOLO)-attention based on YOLOv4 for complex defect types and noisy detection environments in wire and arc additive manufacturing (WAAM), achieving fast and accurate defect detection for WAAM. This method achieved an average accuracy of 94.5% in dynamic images. Kelishadrokhi et al. [27] proposed a new method based on the combination of color and texture features to solve the problem of finding more similar images from a large database. They proposed an extended version of local neighborhood difference pattern (ELNDP) to achieve discriminative features and optimized the color histogram features in the HSV color space to extract color features. This method has better retrieval performance compared to other methods. Xu et al. [28] proposed a new texture structure extraction system. Experimental results show that the algorithm is effective and does not need a priori condition. Zhou et al. [29] proposed a method to extract machine tool defects from high-speed milling workpiece surface images, which reduces the influence of workpiece surface background texture. The application example shows that this method can effectively extract machine tool defects. Su et al. [30] proposed a new edge-preserving texture suppression filter, which uses the joint bilateral filter as a bridge to achieve the dual purposes of texture smoothing and edge-preserving. Isar et al. [31] proposed a two-stage denoising system structure to separate threshold calculation and noise removal so as to improve the denoising performance. Tian et al. [32] designed a lighting method combining plane illumination mode with multiangle illumination mode to automatically detect five kinds of defects by different detection methods.

However, most of the above contour extraction methods have the disadvantages of nonadaptive denoising, a large number of datasets, and texture suppression without edge preservation. Therefore, it is necessary to propose a method that takes into account the weaknesses of the above methods. In order to remove noise and suppress texture simultaneously, while preserving as much detail as possible before scratch detection, we propose an adaptive scratch extraction algorithm framework for detecting scratches with a two-stage system, suppressing interference features. This method is able to detect scratches on the surface of zirconium sheets from the interference of background features effectively. The key of this method is to obtain wavelet decomposition level, threshold, and texture size.

The rest of this article is organized as follows. In Section 2, we first introduce the theoretical part. Then, the experiment and its results are introduced in Section 3. The conclusion and further work are shown in Section 4.

## 2. Methodology

**Figure 1:**
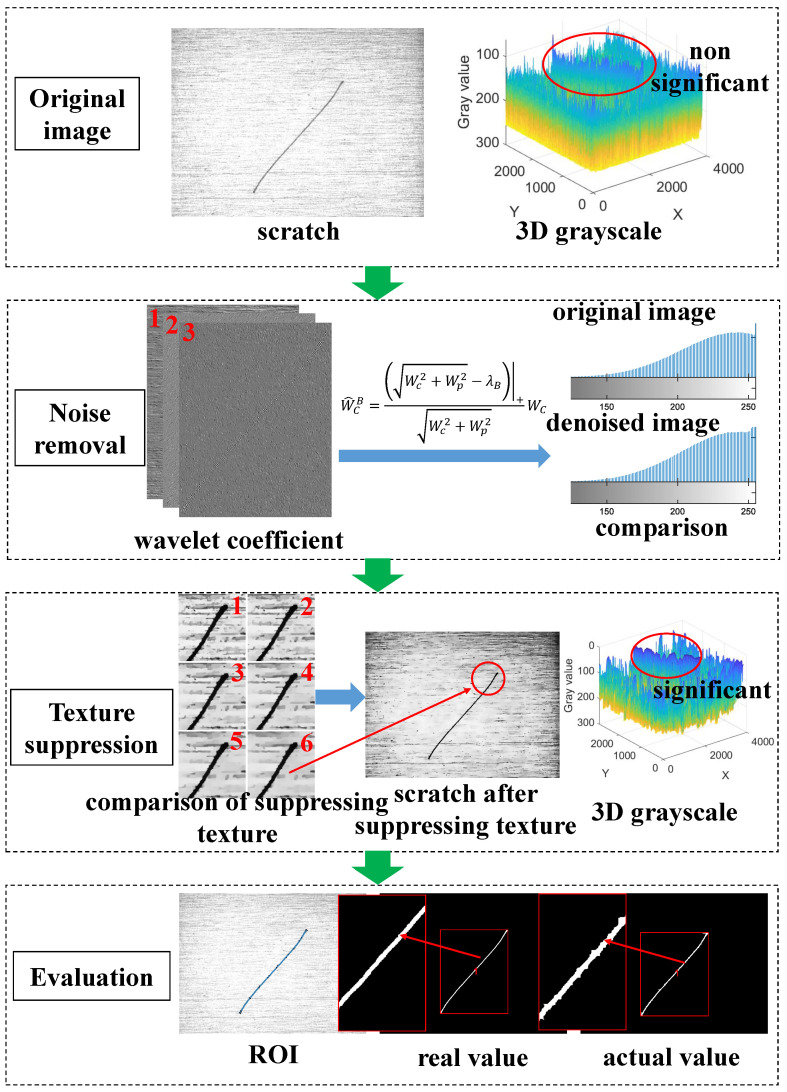
Scheme of algorithm (Six images represented by numbers are the results using different parameters in Texture suppression and the area of red box is scratch region).

Due to the interference of background features, we propose an adaptive scratch extraction framework for the surface of zirconium sheets to detect scratches. At first, this algorithm uses DTCWT function to decompose images with selected wavelet decomposition level, and then designs the optimized adaptive local threshold to improve the ability of removing noise. Secondly, it selects the texture size and then designs a relative total variation enhancement (RTVE) algorithm to suppress texture. Finally, it detects the scratches and quantitatively analyzes these defects from three aspects, e.g., area, length and position deviation. Therefore, this section provides theories related to noise removal and texture suppression, e.g., the objective function for calculating adaptive threshold, RTVE model, parameter selection formula, the evaluation function, etc. The flow chart of the algorithm is shown in Figure 1 and Algorithm 1. In Algorithm 1, $D_{n}$ represents the decomposition level and $T_{ps}$ represents the texture size. The first step is to input the original image and view the 3D grayscale image; the second step is to observe the 3D grayscale images of wavelet coefficients at all levels, select appropriate decomposition level, and remove noise; the third step is to test texture size and then suppress texture; the final step is to extract the region of interest (ROI) and compare it with the results to obtain accuracy.
**Algorithm 1** Scratch extraction algorithm framework.**Input:** *f*: scratch; region of interest (ROI): real value; $D_{n}$ and $T_{ps}$: patameters;**Output:** $f_{c}^{{}^{\prime }}$: image after noise removal and texture suppression; comparison result $[V^{-},D^{-},H^{-},H^{+},D^{+},V^{+}]=\mathrm{DTCWT}\left(f\right)$ **if** 
$D_{n}\in \left[1,4\right]$ 
**then**  ${\hat{W}}_{SST}=\mathrm{SST}\left(V^{-},D^{-},H^{-},H^{+},D^{+},V^{+}\right)$  $N_{f}=f-\mathrm{IDTCWT}\left({\hat{W}}_{SST}\right)$  ${\hat{W}}_{c}^{B}=\mathrm{BTS}\left(f\right)$  $f_{d}=\mathrm{IDTCWT}\left({\hat{W}}_{c}^{B}\right)$ **end if** **if** 
$T_{ps}\in \left[1,6\right]$ 
**then**  $f_{c}^{{}^{\prime }}=\mathrm{RTVE}\left(f_{d}\right)$ **end if** Comparison of results and ROI **return** $f_{c}^{{}^{\prime }}$ and comparison result


### 2.1. Adaptive Noise Removal in Wavelet Domain

**Property 1.**  *The two types of double hook functions are described as*

(1) $$\begin{array}{c} y=ax+\frac{b}{x}\left(ab>0\right), \end{array}$$

*where a and b are constants; a,b≠0. Normally, a=b=1.*

(2) $$\begin{array}{c} y=ax+\frac{b}{x}\left(ab<0\right), \end{array}$$

**Figure 2:**
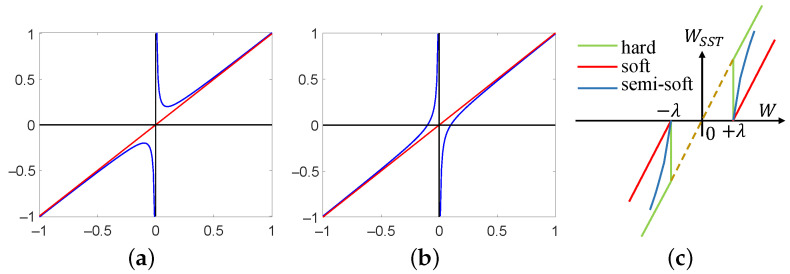
Images of three functions. (**a**) Prototype I (The red line represents the asymptote, and the blue curve represents Equation (1)). (**b**) Prototype II (The red line represents the asymptote, and the blue curve represents Equation (2)). (**c**) Threshold functions.

*where, normally, a=1,b=−1. The double hook function has two asymptotes, one vertical asymptote and one oblique asymptote. Equation (1) has an oblique asymptote and has no intersection with the x axis. Equation (2) is different from Equation (1) because it intersects with the x axis. Equations (1) and (2) are shown in Figure 2.*

**Property 2.**  *The hard threshold function and soft threshold function are described as [33,34]*

(3) $$\begin{array}{c} {\hat{W}}_{HT}=\left\{\begin{array}{cc} 0, & |W|\leq \lambda \\ W, & |W|>\lambda \end{array}\right., \end{array}$$

(4) $$\begin{array}{c} {\hat{W}}_{ST}={sgn\left(|W|-\lambda \right)}_{+}, \end{array}$$

*where λ is threshold, W is wavelet coefficients, and HT and ST are hard threshold function and soft threshold function, respectively. Equations (3) and (4) are shown in Figure 2c.*

We propose an improved threshold model according to Equation (2) of Property 1, which takes into account the advantages of both soft and hard threshold functions. The semisoft threshold formula is described as

(5) $$\begin{array}{c} {\hat{W}}_{SST}=\left\{\begin{array}{cc} 0 & ,|W|\leq \lambda_{A}\\ sgn\left(W\right) [\left|W\right|-\lambda_{A}^{2}/\left|W\right|] & ,\left|W\right|>\lambda_{A} \end{array}\right., \end{array}$$

where $\lambda_{A}$ is threshold, sgn represent step function, and ${\hat{W}}_{SST}$ is the result of filtering. Furthermore, $\lambda_{A}$ is described as

(6) $$\begin{array}{c} \lambda_{A}=\sqrt{2}\frac{{\hat{\sigma }}_{n}^{2}}{\hat{\sigma }}, \end{array}$$

where ${\hat{\sigma }}_{n}^{2}$ is estimated variance for the noise component, and $\hat{\sigma }$ is estimated variance for the noiseless component.

In the first stage of removing noise, the soft threshold function or hard threshold function is usually used to extract noise components of each sub-band. However, from Figure 2c, the wavelet coefficients processed by the hard threshold function generate jump points at $\pm \lambda_{A}$, do not have the smoothness of the original information, and oscillation occurs after reconstruction. Therefore, this disadvantage can lead to sudden changes in the pixels on the scratch image, i.e., there is a significant difference in the grayscale values of some adjacent pixels. The wavelet coefficients processed by the soft threshold function have a fixed deviation compared to the hard threshold function, i.e., it directly affects the similarity between the reconstructed coefficients and the real coefficients. Therefore, this disadvantage can cause the overall shift of pixel grayscale values on the scratch images, resulting in a blurry effect.

From Figure 2c, we can intuitively see the shapes and features of the hard threshold function and the soft threshold function. Among them, the red line represents the soft threshold function, the green line represents the hard threshold function, and the blue curve represents the semisoft threshold function. Based on the characteristics of Figure 2c, the common feature of both functions can be obtained, which is that they are parallel to the line ${\hat{W}}_{SST}=W$. Therefore, considering the drawbacks of both functions, we need an asymptotic semisoft threshold function to compensate for the discontinuity of the hard threshold function and the deviation of the soft threshold function, i.e., it can eliminate sudden changes in pixel grayscale values and weaken blurring effects. Since the line ${\hat{W}}_{SST}=W$ belongs to the oblique asymptote, and Equation (2) has this feature, we construct the formula shown in Equation (5).

In order to verify that Equation (5) converges to a straight line ${\hat{W}}_{SST}=W$, we assume two scenarios. When $W>\lambda_{A}$, i.e., *W* is also greater than 0, according to Equation (5), the transformed formula can be described as

(7) $$\begin{array}{c} \frac{{\hat{W}}_{SST}}{W}=1-\frac{\lambda_{A}^{2}}{W^{2}}, \end{array}$$

by applying the limit to Equation (7), the result can be obtained as

(8) $$\begin{array}{c} \lim_{W\to +\infty }\frac{{\hat{W}}_{SST}}{W}=1. \end{array}$$

When $W<-\lambda_{A}$, i.e., *W* is also less than 0, according to Equation (5), the transformed formula can be described as

(9) $$\begin{array}{c} \frac{{\hat{W}}_{SST}}{W}=1-\frac{\lambda_{A}^{2}}{W^{2}}, \end{array}$$

by applying the limit to Equation (9), the result can be obtained as

(10) $$\begin{array}{c} \lim_{W\to -\infty }\frac{{\hat{W}}_{SST}}{W}=1. \end{array}$$

Therefore, based on the above results, we have the following conclusion:

(11) $$\begin{array}{c} \lim_{W\to \infty }\left({\hat{W}}_{SST}-W\right)=0. \end{array}$$

We can see that Equation (5) has only one oblique asymptote ${\hat{W}}_{SST}=W$. As |W| continues to increase, the value of $\lambda_{A}^{2}/\left|W\right|$ gradually decreases and eventually approaches *W*, resulting in a gradual decrease in the deviation between the estimated wavelet coefficients and the actual wavelet coefficients, overcoming the shortcomings of the two threshold functions.

It is worth noting that, in the first stage, we use the square directional window to calculate the variance as we need to extract as much noise as possible from images; the square window can meet the requirements. In the second stage, we use elliptical directional windows to calculate the variance to preserve as much detail as possible while denoising. However, elliptical windows have poor directionality and are bulky in volume.

Therefore, in the second stage of removing noise, we use a parameters-adjusted elliptical direction window with smaller size and more monotonic direction for calculating local threshold based on the elliptical window prototype [35]. The formula is defined as

(12) $$\begin{array}{c} \begin{array}{c} W\left(\theta \right)=\left\{\left.\left(m,n\right)\right|{\left(m\sin\theta +n\cos\theta \right)}^{2}/3^{2}+{\left(m\cos\theta -n\sin\theta \right)}^{2}/1.5^{2}\leq 1\right\}, \end{array} \end{array}$$

where $\theta \in [-\pi ,\pi ]$ is the principal axis direction; when it is $\pm 15{}^{\circ }$, $\pm 45{}^{\circ }$ or $\pm 75{}^{\circ }$, it corresponds to the elliptical window of six high-frequency sub-bands, and *m* and *n* are coordinates of pixels. Finally, the image of removing noise is obtained by inverse transformation.

### 2.2. Texture Suppression and Edge Preservation

We propose one RTVE model, which can maintain the defect contour while suppressing texture. Using RTVE to suppress texture of the image $f_{d}$, the result $f_{c}^{{}^{\prime }}$ is defined as

(13) $$\begin{array}{c} f_{c}^{{}^{\prime }}=\min_{S}\left(\frac{1}{2}{∥f_{d}-S∥}_{2}^{a}+\lambda_{t}\sum_{p}F_{RTVE}\left(p\right)\right), \end{array}$$

where ${∥f_{d}-S∥}_{2}^{a}$ enhances the contrast of the image $f_{d}$ and is defined as

(14) $$\begin{array}{c} {∥f_{d}-S∥}_{2}^{a}=\left\{\begin{array}{c} P_{1\%}^{lowest}=\mathrm{minPixel}\\ P_{1\%}^{highest}=\mathrm{maxPixel} \end{array}\right., \end{array}$$

where $P_{1\%}^{lowest}$ denotes the minimum 1% pixels set as the minimum pixel value and $P_{1\%}^{highest}$ denotes the maximum 1% pixels set as the maximum pixel value. Furthermore, $F_{RTVE}\left(p\right)$ is described as

(15) $$\begin{array}{c} \begin{array}{c} F_{RTVE}\left(p\right)=D_{x}^{E}\left(p\right)/\left(L_{x}^{E}\left(p\right)+\varepsilon \right)+D_{y}^{E}\left(p\right)/\left(L_{y}^{E}\left(p\right)+\varepsilon \right), \end{array} \end{array}$$

where ε is a small positive number, avoiding zero denominator, $D_{x}^{E}\left(p\right)$ and $D_{y}^{E}\left(p\right)$ denote the total variations of the window for pixel *p* in the x and y directions, respectively, and $L_{x}^{E}\left(p\right)$ and $L_{y}^{E}\left(p\right)$ are the inherent variations of the window for pixel *p* in x and y directions, respectively.

All parameters of Equation (15) are described, and $D_{x}^{E}\left(p\right)$ is described as

(16) $$\begin{array}{c} D_{x}^{E}\left(p\right)=\left(1/W_{p}\right)\sum_{q\in R\left(p\right)}g_{p,q}^{E}\cdot \left|{\left(\partial_{x}S\right)}_{q}\right|, \end{array}$$

and $D_{y}^{E}\left(p\right)$ is described as

(17) $$\begin{array}{c} D_{y}^{E}\left(p\right)=\left(1/W_{p}\right)\sum_{q\in R\left(p\right)}g_{p,q}^{E}\cdot \left|{\left(\partial_{y}S\right)}_{q}\right|, \end{array}$$

$L_{x}^{E}\left(p\right)$ is described as

(18) $$\begin{array}{c} L_{x}^{E}\left(p\right)=\left|\left(1/W_{p}\right)\sum_{q\in R\left(p\right)}g_{p,q}^{E}\cdot {\left(\partial_{x}S\right)}_{q}\right|, \end{array}$$

and $L_{y}^{E}\left(p\right)$ is described as

(19) $$\begin{array}{c} L_{y}^{E}\left(p\right)=\left|\left(1/W_{p}\right)\sum_{q\in R\left(p\right)}g_{p,q}^{E}\cdot {\left(\partial_{y}S\right)}_{q}\right|, \end{array}$$

$W_{p}$ is described as

(20) $$\begin{array}{c} W_{p}=\sum_{q\in R}g_{p,q}^{E}, \end{array}$$

$g_{p,q}^{E}$ is described as

(21) $$\begin{array}{c} \begin{array}{c} g_{p,q}^{E}\propto \left\{exp\left\{-\left({\left(x_{p}-x_{q}\right)}^{2}+{\left(y_{p}-y_{q}\right)}^{2}\right)/2\sigma^{2}\right\}\cdot exp\left\{-{ [I\left(i,j\right)-I\left(m,n\right)]}^{2}/{2\sigma }_{r}^{2}\right\}\right\}, \end{array} \end{array}$$

where *i* and *j* are the abscissa and ordinate of the central pixel, m and n are the abscissa and ordinate of the input pixel, $I\left(i,j\right)$ is the value of the central pixel, and $I\left(m,n\right)$ is the value of the input pixel. In addition, $g_{p,q}^{E}$ denotes the product of two weights.

Usually, we use relative total variation (RTV) to suppress texture [28]. Furthermore, the objective function of RTV is defined as [28]

(22) $$\begin{array}{c} f_{c}=\min_{S}\left(\frac{1}{2}{∥f_{d}-S∥}_{2}+\lambda_{t}\sum_{p}F_{RTV}\left(p\right)\right), \end{array}$$

where $\lambda_{t}$ is the weight of the regular item, and $F_{RTV}\left(p\right)$ is defined as

(23) $$\begin{array}{c} \begin{array}{c} F_{RTV}\left(p\right)=D_{x}\left(p\right)/\left(L_{x}\left(p\right)+\varepsilon \right)+D_{y}\left(p\right)/\left(L_{y}\left(p\right)+\varepsilon \right). \end{array} \end{array}$$

When the RTV method is applied to the surface of metal materials with structural texture, it is difficult for RTV to completely suppress the texture background while ensuring that the small size defects are not lost, because RTV only considers the weight of spatial distance. Equation (15) reflects on the calculation of two weights to compensate for the shortcomings of RTV. Not only is the influence of spatial distance weight considered, but the gray difference between the central pixel and other pixels in the neighborhood is also taken as the basis for calculating another weight.

**Figure 3:**
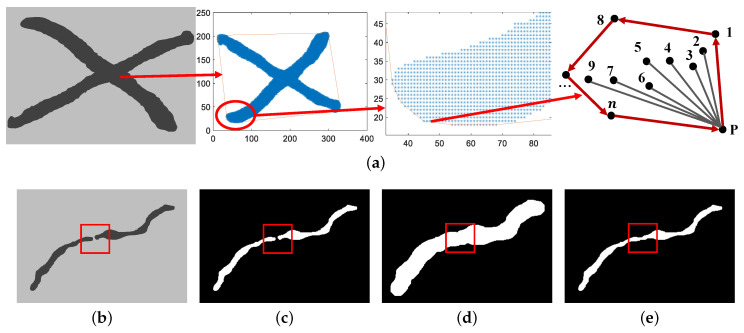
Two connection methods. (**a**) Convex hull of scratches (Points represented by numbers are different pixels). (**b**) Original image (The area of red box indicates the location of the fracture). (**c**) Binary image. (**d**) Expansion of scratches. (**e**) Corrosion of scratches.

After the end of preprocessing, it is necessary to ensure the integrity of scratches based on the convex hull and close operation to connect the broken part. The principle of convex hull is shown in Figure 3a. The expansion and corrosion of close operation are shown in Figure 3b–e.

Compared with the manually extracted ROI, the scratch extraction of the real image is evaluated by area, length, and position deviation. The comprehensive accuracy is the arithmetic mean of the first two accuracy. Furthermore, it is described as

(24) $$\begin{array}{c} \mathrm{Total}=\frac{1}{2}\left(\mathrm{area}+\mathrm{length}\right). \end{array}$$

The position deviation is expressed by the Euclidean distance between the centroids,

(25) $$\begin{array}{c} d_{e}=\sqrt{{\left(x_{1}-x_{2}\right)}^{2}+{\left(y_{1}-y_{2}\right)}^{2}}, \end{array}$$

where $x_{1}$ and $y_{1}$ represent the centroid of the real value, $x_{2}$ and $y_{2}$ represent the centroid of the actual value, and $d_{e}$ represents the distance difference between the two. As long as the distance difference is less than 100 pixels, we consider this distance to be qualified.

## 3. Experiments

**Figure 4:**
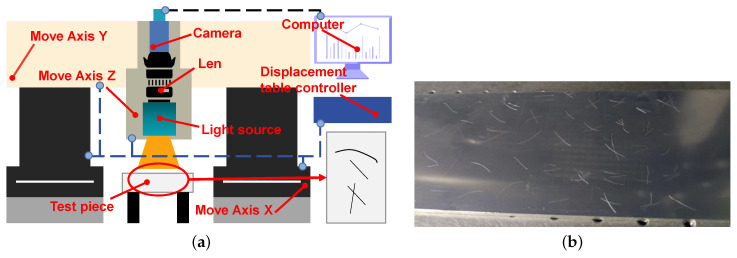
System structure and test piece. (**a**) System structure. (**b**) Test piece.

As shown in Figure 4, the scratch images used in this paper are collected by the machine vision system, which is composed of a three-dimensional motion device and a pixel resolution of 4024 × 3036 industrial camera, a white light source, a computer, and a test piece. Three-dimensional space movement of the camera and light source is realized through control software to ensure the collection of scratch images.

**Figure 5:**
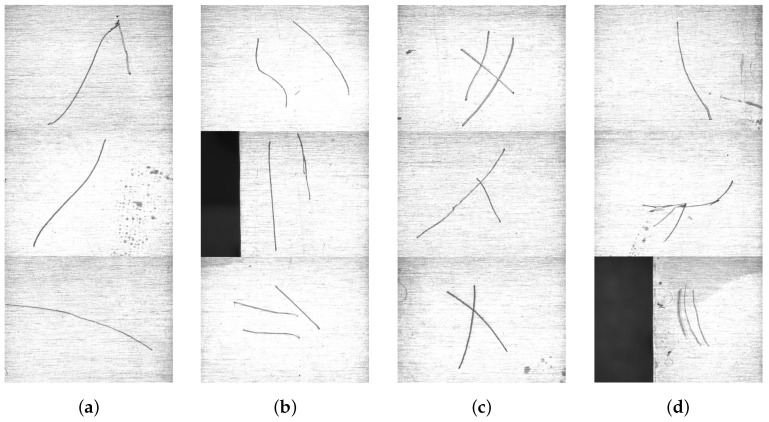
Different types of scratches. (**a**) Three types of single scratch. (**b**) Three types of multiple scratches. (**c**) Three types of cross scratch. (**d**) Three types of other scratches.

The surface images of zirconium sheets are shown in Figure 4b. Among them, aluminum plate is used to replace zirconium sheet. There are four kinds of scratch images in this paper, including single scratch, multiple scratches, cross scratch, and other scratches, as shown in Figure 5. It is noteworthy that the size of these images is processed later due to the need of wavelet transform. Before collecting images, we use alcohol to wipe the surface of the sample due to interference from other things. In addition, before the experiment, we test the light intensity. Lighting has a significant impact on the imaging quality. Different lighting methods have different effects on the detection object; for example, diffuse light sources can cause the detection surface to emit light, requiring special light sources to solve the problem of reflection. Therefore, choosing the appropriate light source and appropriate lighting intensity is crucial for nondestructive testing technology based on machine vision. By analyzing the surface properties of the sample and investigating the types and application scenarios of light sources, we design a machine vision inspection system with a high-precision camera and a coaxial light source as the core due to the high reflectivity and complex texture on the surface of the detected object. Firstly, we use coaxial light sources to reduce the impact of reflected light due to the inability of ordinary light sources to suppress surface reflection. Secondly, we choose the appropriate lighting intensity through experiments to ensure the integrity of the scratch area while minimizing the texture as much as possible. We use the algorithm proposed in this article to test scratch images obtained from different lighting intensities and compare them with other algorithms. It is a good choice to increase the light intensity as much as possible while ensuring the integrity of scratches according to the data. Therefore, choosing a value within the appropriate range of light intensity is the correct choice.

**Figure 6:**
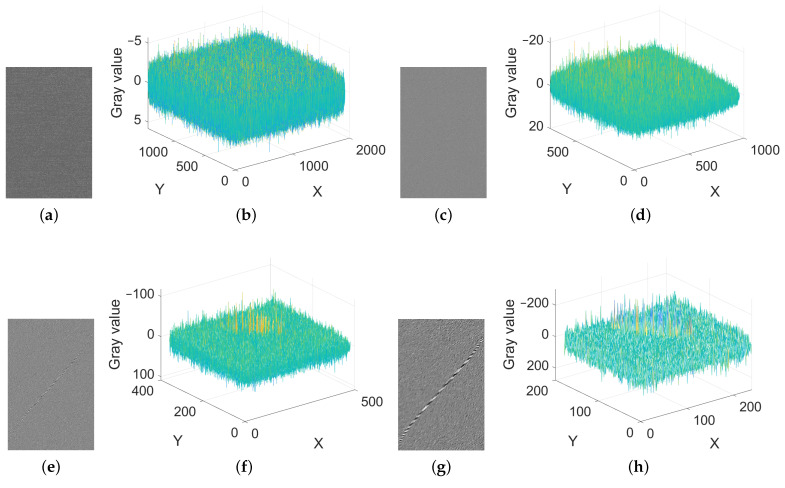
Selection of decomposition level (cropped). (**a**,**b**) The wavelet coefficient of the first level at 45° and its 3D grayscale. (**c**,**d**) The wavelet coefficient of the second level at 45° and its 3D grayscale. (**e**,**f**) The wavelet coefficient of the third level at 45° and its 3D grayscale. (**g**,**h**) The wavelet coefficient of the fourth level at 45° and its 3D grayscale.

Before the scratch detection, it is necessary for inputs to remove noise and suppress texture. The first half of Section 3 introduces the content of noise removal and texture suppression, respectively. From Figure 6, observing the change trend of wavelet coefficients at 45° from first level to fourth level and their 3D grayscale due to significant characteristics of diagonal coefficients, we can summarize the following conclusions. At first, from Figure 6a–d, the wavelet coefficients of the first and second levels cannot reflect detailed information. Second, from Figure 6g–h, the wavelet coefficients of the fourth level will be distorted due to the loss of pixels. Finally, from Figure 6e,f, the wavelet coefficients of the third level can retain rich scratch information while also reflecting necessary background interference. In the process of decomposing the levels from 1 to n, the details of scratches move from blurry to clear, and then to blurry. In addition, when the decomposition level is 3, the scratch details are most obvious. Therefore, we choose the third decomposition level, i.e., $D_{n}$ is 3.

**Figure 7:**
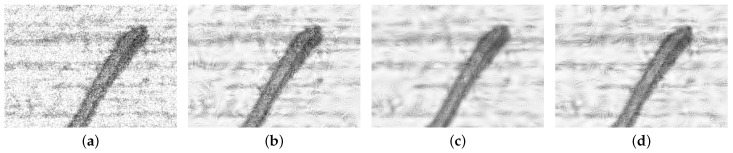
Comparison of denoising methods. (**a**) Noise added. (**b**) Hard threshold processing. (**c**) Soft threshold processing. (**d**) Semisoft threshold processing.

As shown in Figure 7, the image pixels after hard thresholding appear abrupt, and the pixels after soft thresholding are blurry and smooth, while the pixels after semisoft thresholding are between the two thresholds, which not only weakens the sudden changes in pixel values but also maintains smoothness.

**Figure 8:**
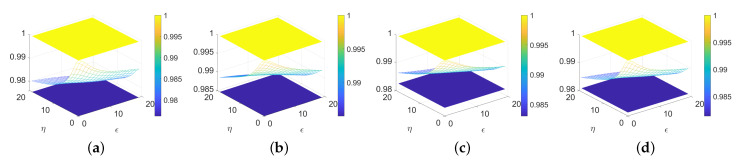
Texture size. (**a**) Single scratch I. (**b**) Multiple scratches I. (**c**) Cross scratch I. (**d**) Other scratches I.

On the basis of removing noise, we need to suppress structural texture. Firstly, the parameter selection method is used to calculate the texture size. The texture size is determined by the height shown in Figure 8. By analyzing Figure 8, we estimate the texture size and select the parameter in 0–6. When the autocorrelation coefficient is greater than 0.01, the texture size is roughly between 3 and 6. Furthermore, when the autocorrelation coefficient is less than 0.01, the texture size is roughly between 0 and 3. Therefore, we can obtain $T_{ps}$ based on texture size.

The height difference between the yellow plane and the blue plane indicates the texture size; thus, it can be seen from Figure 8 that the autocorrelation coefficients are 0.0238, 0.0150, 0.0170, and 0.0187, respectively. Therefore, the texture size can be roughly determined to be over 3.

**Figure 9:**
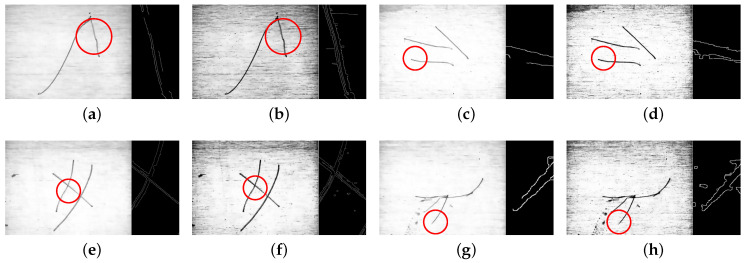
Comparison of texture suppression methods (The black image is an enlargement of the area in the red circle). (**a**,**b**) Single scratch I. (**c**,**d**) Multiple scratches III. (**e**,**f**) Cross scratch I. (**g**,**h**) Other scratches II.

Then, the results obtained by using RTVE are compared with those obtained by RTV. Figure 9 shows the surface of the scratch images after suppressing texture. Figure 9a,c,e,g are processed by RTV. Furthermore, Figure 9b,d,f,h are processed by RTVE. By analyzing Figure 9, we can see that the integrity of the scratch edge is higher after being processed by the RTVE algorithm. On the contrary, the scratch area processed by RTV always loses some edges, indicating that the RTVE algorithm proposed in this paper has advantages that RTV does not have.

**Figure 10:**
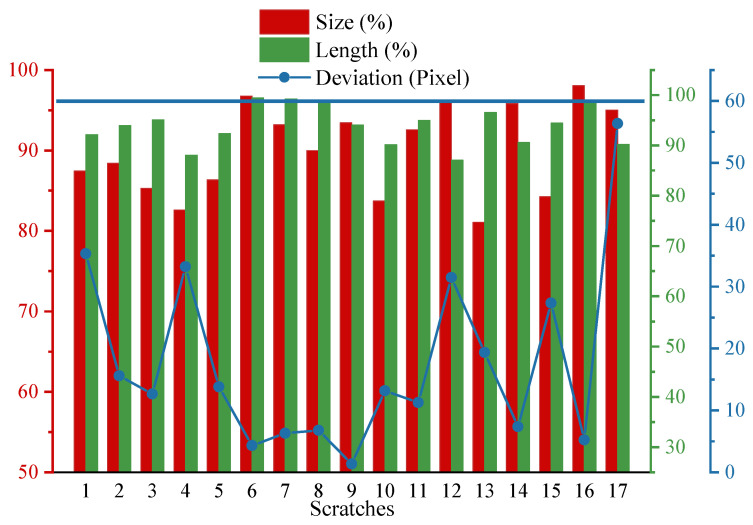
Comparison of different scratches.

**Figure 11:**
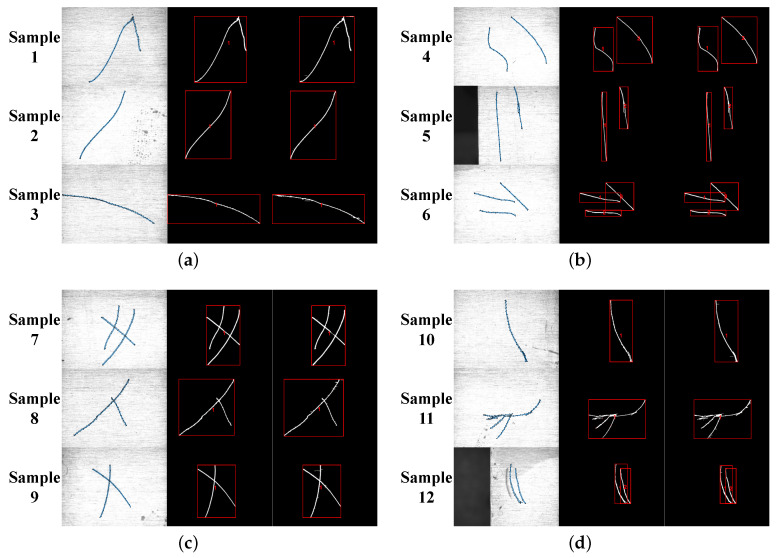
The contrast of extracted scratches (From left to right, they are ROI, real value, and actual value; The area of red box represents the scratch region). (**a**) Single scratch. (**b**) Multiple scratches. (**c**) Cross scratch. (**d**) Other scratches.

The proposed noise removal and texture suppression methods are used to process the real scratch images. Before detecting scratches, image preprocessing is required. In order to quantitatively evaluate the performance of the algorithm proposed in this paper, the error with the real value is calculated from three aspects of area, length, and position deviation, using the manually extracted region of interest as real value. The results are shown in Figure 10 and Table 1. The red boxes of Figure 11a–d are the area where the scratch is located.

From Table 1 and Figure 11, we know that there are four types of scratches, each with three types, totaling 17 scratches. The real value indicates ROI, and scratches are manually extracted from the scratch images. The actual value represents the results obtained by the framework proposed in this paper. By comparative analysis, we can see that the accuracy of this algorithm is greater than 85% and the algorithm has more advantages in terms of accuracy.

The framework proposed in this article is compared with current scratch detection algorithms, and the results are shown in Table 2. These algorithms are snake based on edge, fuzzy edge, log, K-means, fuzzy c-means, and method of combining superpixels with k-means (the original Canny is not included because using only Canny cannot remove texture) [36]. The results show that the algorithm proposed in this article has better performance, can obtain more complete scratches, and is superior to other gradient algorithms. Compared with log, the proposed algorithm is more accurate and stable. The edge-based snake algorithm requires masks, and the number of iterations is difficult to determine, resulting in a wide fluctuation range of results. Although the texture is suppressed, the fuzzy edge algorithm will still recognize the residual texture as an edge of scratches, resulting in incorrect results. Compared with image segmentation algorithms, the algorithm proposed in this article can accurately obtain complete scratch contours.

## 4. Conclusions

This study proposes a scratch detection framework for zirconium sheets with surface interference. As can be seen from the previous text, this framework includes two parts: noise removal and texture suppression. An adaptive threshold algorithm based on DTCWT is proposed for denoising. At the same time, an optimized texture suppression algorithm based on RTVE is proposed for suppressing texture. Finally, an optimized Canny algorithm is used to detect scratch contours, and the result is compared with the real value.

This surface scratch detection solution is able to remove noise and suppress texture, solving the problem of background interference. The experimental results show that the comprehensive accuracy is greater than 85%, and the framework can effectively improve the accuracy of scratch detection. This optimized scratch detection algorithm framework is equivalent to a semiadaptive local threshold method, which is not only suitable for metal parts but also for other materials with similar surface, such as tile scratches. In the future work, complete adaptive scratch detection can be realized through threshold calculation, morphological processing and image enhancement, so that defects of different degrees and types can be detected.

## Figures and Tables

**Table 1:** The results of scratches.

Scratches	Real Value			Actual Value			Total
	Area (pixel)	Length (pixel)	Centroid	Area (pixel)	Length (pixel)	Centroid	
1	132,822	5450	2189.5 1335.7	149,500	5023	2206.2 1304.6	89.81%
2	86,849	3790	1508.8 1479.7	96,926	4019	1504.5 1482.7	91.18%
3	90,674	4370	1754.9 1470.4	104,000	4155	1762.0 1482.0	90.19%
4	41,853	2026	1549.6 1532.2	49,132	2268	1537.7 1501.2	85.34%
5	47,642	2535	2858.0 1150.9	54,146	2729	2848.9 1140.5	89.35%
6	53,173	2608	1622.7 1445.0	54,897	2622	1623.2 1449.3	98.12%
7	42,575	2016	2363.8 736.8	45,472	2031	2365.0 743.0	96.23%
8	41,083	1663	1449.4 1204.0	45,196	1647	1442.7 1203.0	94.52%
9	36,675	1442	1602.8 1752.6	39,084	1528	1601.6 1752.0	93.74%
10	34,528	1775	2226.1 1167.2	40,146	1600	2217.0 1157.7	86.94%
11	197,936	7545	2047.4 1595.7	212,650	7924	2039.0 1603.2	93.78%
12	123,206	5648	1657.4 1518.0	127,983	4917	1678.9 1495.0	91.67%
13	115,621	4607	1732.4 1489.2	137,520	4765	1735.0 1470.0	88.82%
14	77,264	3532	2234.3 1682.2	80,494	3199	2240.3 1686.5	93.20%
15	125,567	6155	1952.5 1795.5	145,347	5814	2013.1 1789.1	89.36%
16	44,494	1988	2198.6 1498.7	45,367	2012	2196.7 1503.6	98.42%
17	39,005	1520	2390.4 1458.8	37,062	1669	2403.5 1513.6	92.61%

**Table 2:** Comparison of methods.

Sample	Single		Muti		Cross		Other	
	Area	Length	Area	Length	Area	Length	Area	Length
The proposed	87.44%	92.17%	84.48%	90.21%	92.57%	94.98%	84.25%	94.46%
Log	72.33%	90.50%	69.53%	82.03%	87.21%	98.69%	70.15%	92.46%
Snake-based on edge	91.02%	65.43%	70.39%	75.89%	81.50%	61.56%	63.29%	57.68%
Fuzzy edge	78.77%	98.06%	71.64%	73.75%	86.50%	97.16%	63.59%	96.88%
K-means	93.11%	92.72%	97.39%	79.85%	62.96%	88.69%	77.22%	94.88%
Fuzzy c-means	93.83%	81.85%	98.21%	84.65%	84.10%	77.84%	97.52%	80.00%
K-means and superpixels	95.29%	79.94%	88.07%	62.87%	65.37%	94.88%	82.02%	72.85%

## Data Availability

The data are not publicly available due to personal privacy.
